# Comparison of contralateral muscle excitation in proximal versus distal muscles in the upper extremities

**DOI:** 10.3389/fnhum.2025.1718126

**Published:** 2025-12-17

**Authors:** Alexander Nynes, David McGhie, Morten A. Aune, Tore Kristian Aune

**Affiliations:** Department of Sport Science, Sport and Human Movement Science Research Group (SaHMS), Nord University Levanger, Norway, Levanger, Norway

**Keywords:** proximal vs distal, motor coordination and control, contralateral muscle excitation, neural crosstalk, corticospinal integration, surface electromyography (sEMG)

## Abstract

**Introduction:**

The purpose of the study was to investigate potential differences in contralateral muscle excitation in proximal versus distal muscles in the upper extremities. Based on the different neuroanatomical and neurophysiological constraints in the central part of the neural system for proximal and distal muscles, it was hypothesized that contralateral muscle excitation (CME) would be higher for proximal than distal muscles.

**Methods:**

Thirteen university students participated in this study. The participants performed isometric flexion movements with the shoulder and index finger of the dominant arm at four different relative force levels (25, 50, 75, 100%). Force was measured with a force transducer connected to the index finger and elbow on the dominant arm. Muscle excitation was measured using sEMG placed on the flexor carpi radialis (distal condition) and the anterior deltoid (proximal condition) on the non-dominant arm.

**Results:**

CME was observed in both proximal and distal muscles, with proximal muscles displaying significantly higher CME at higher force levels (50, 75, and 100%). In the proximal condition, contractions with the dominant anterior deltoid were associated with a progressive increase in CME across force levels in the contralateral homologous muscle. In contrast, for the distal condition (flexor carpi radialis), CME changes were only evident when comparing the lowest and highest force levels.

**Conclusion:**

The results are in coherence with the differences in neuroanatomical and neurophysiological constraints for bilateral communication for proximal versus distal muscles. The present results encourage further neurophysiological studies using direct brain activity measures to explore the potential link between the differences in bilateral communication and CME for proximal versus distal muscles.

## Introduction

Several studies have shown that, during unilateral contractions, muscle excitation is not restricted to only the specific activated ipsilateral muscles but also occurs in homologous contralateral muscles (Abreu et al., 2015; Carolan and Cafarelli, 1992; Carroll et al., 2006; Cernacek, 1961; Cincotta and Ziemann, 2008; Heming et al., 2019; Munn et al., 2004; Post et al., 2008; Zijdewind et al., 2006; Zijdewind et al., 1998). Various terms have been employed to describe this phenomenon (e.g., associated activity, mirror movement, cross-over effect, motor irradiation, contralateral learning, inter-limb transfer, and bilateral activity). In the present study, the term contralateral muscle excitation (CME) refers to the unintended muscular excitation observed in the contralateral homologous muscles. Typically, this phenomenon happens involuntarily and often goes unnoticed by the individual. In adults, research has shown a positive correlation between contralateral muscle excitation and the contraction in the targeted muscle (Abreu et al., 2015; Armatas et al., 1994; Bodwell et al., 2003; Dimitrijevic et al., 1992; Shinohara et al., 2003; Tsuchiya et al., 2018; Valdes et al., 2021). Specifically, increasing the force of the contraction in the targeted muscle is closely linked to the excitation observed in contralateral muscles (Armatas et al., 1994; Bodwell et al., 2003; Dimitrijevic et al., 1992; Shinohara et al., 2003; Todor and Lazarus, 1986).

From a theoretical perspective, the specific mechanism responsible for contralateral muscle excitation is still not fully understood (Carroll et al., 2006; Green and Gabriel, 2018). Therefore, investigating the mechanisms behind this phenomenon is important for gaining more knowledge on neurophysiological constraints that affect both unilateral and bilateral coordination and control of the upper extremities. Previous research on unilateral strength training has suggested cross-activation and neural crosstalk as two main plausible explanations for CME (Green and Gabriel, 2018; Lee and Carroll, 2007; Ruddy and Carson, 2013). The cross-activation theory posits that unilateral activity stimulates both the ipsilateral and contralateral cortex, leading to neural adaptations in both hemispheres. In contrast, the neural crosstalk theory suggests that unilateral effects on contralateral muscles arise from interhemispheric communication, causing adaptations in untrained muscles (Carson, 2005; Lee and Carroll, 2007; Ruddy and Carson, 2013), and that interhemispheric communication between associated motor areas can be both faciliatory and inhibitory (Aune et al., 2024; Škarabot et al., 2016). These neurophysiological explanations are not necessarily mutually exclusive.

Research has demonstrated that neural crosstalk is an important organismic constraint in interhemispheric communication, and can facilitate both excitatory and inhibitory neural drive to the contralateral muscles during unilateral motor tasks (Armatas et al., 1994; Bannatyne et al., 2003; Delwaide and Pepin, 1991; Ferbert et al., 1992; Hortobágyi et al., 2003; Hübers et al., 2008; Kinsbourne, 1975; Liuzzi et al., 2011; Serrien and Brown, 2002; Takeuchi et al., 2011). In addition, an increase or decrease in the neural drive to the contralateral hemisphere during both unilateral and bilateral motor tasks depends on the characteristics of the motor task performed (Khodiguian et al., 2003; Oda and Moritani, 1995; Taniguchi et al., 2001). Further, the potential for transfer of neural information between the left and right side of the body differs between proximal and distal muscles. Specifically, at the cortical level, the representations of proximal muscles exhibit a higher density of transcallosal projections between the two hemispheres than the more lateralized representations of distal muscles (Brodal, 2010; Gould et al., 1986; Jankowska et al., 2005; Jenny, 1979; Pandya and Vignolo, 1971; Pierrot-Deseilligny and Burke, 2005; Rouiller et al., 1994). This structural difference facilitates more extensive bilateral communication for proximal muscles compared to distal muscles. The enhanced potential for bilateral communication at the cortical level is complemented by findings at the spinal level. Studies by Gould et al. (1986), Pandya and Vignolo (1971), and Jenny (1979) revealed that commissural interneurons at the spinal level exhibit higher connectivity for proximal muscles compared to distal muscles, increasing the potential for neural crosstalk in proximal muscles. In addition, the neural pathways from the motor cortex to the muscles differ for proximal and distal muscles. Specifically, proximal muscles are primarily innervated through polysynaptic connections in the ventromedial corticospinal tract, whereas distal muscles are innervated through monosynaptic connections in the lateral corticospinal tract (Brodal, 2010; Kuypers, 1978; Palmer and Ashby, 1992). Thus, the higher proportion of polysynaptic connections between the motor cortex and proximal muscles indicates a greater likelihood of neural crosstalk for proximal compared to distal muscles (Aune et al., 2020; Aune et al., 2021; Aune et al., 2017; Wang et al., 2022a).

From an applied perspective, researchers have suggested that the occurrence of CME could have a significant impact on functional capability in both unilateral and bilateral movements (Carroll et al., 2006; Munn et al., 2004; Wang et al., 2022a). In daily activities, CME can enhance stability and support during fine motor tasks requiring precision, such as those involving hand-eye coordination (Armatas et al., 1994; Cincotta et al., 2003; Cincotta and Ziemann, 2008; Mayston et al., 1999; McDowell and Wolff, 1997). Further, the presence of CME is especially observed in unilateral strength training studies showing improvements in strength in the untrained contralateral limb during a unilateral training intervention of the ipsilateral limb (Carroll et al., 2006; Munn et al., 2004). Thus, knowledge of the origin of CME can help coaches design exercises that enhance bilateral transfer, potentially improving performance and reducing the risk of injuries. Additionally, understanding the mechanisms behind CME can inform the design of effective rehabilitation programs for individuals recovering from unilateral injuries or neurological disorders such as strokes (Green and Gabriel, 2018; Lodha et al., 2012; Rose and Winstein, 2004; Sarabon et al., 2020; Stewart et al., 2006). This knowledge is particularly valuable in clinical practice for stroke patients, where restoring function in muscles around both shoulders and elbows (proximal) and wrists and fingers (distal) is essential (Pollock et al., 2014; Wang et al., 2022a).

The purpose of the present study was to investigate CME in homologous proximal and distal muscles in the non-dominant arm during an isometric flexion task with the dominant arm. It was hypothesized that CME in the non-dominant arm would be higher for proximal muscles compared to distal muscles. Further, it was hypothesized that CME would increase proportionally with the relative force exerted by the dominant arm for both proximal and distal muscles.

## Materials and methods

### Participants

Thirteen university students (three women and ten men, with a mean ± SD age of 26.6 ± 8.4 years) with no known neuromuscular disabilities were included in the study. Prior to the study, all participants gave their written consent to participate. As indicated by the Edinburg Handedness Inventory test (Oldfield, 1971), four participants were left-handed and nine were right-handed. The protocol was evaluated and approved by the Norwegian Agency for Shared Services in Education and Research (SIKT; Project number: 152360) and performed in accordance with the Declaration of Helsinki.

### Task

The task consisted of pulling a firmly mounted S-type push-pull load cell with constrained maximal voluntary isometric contraction (MVIC) and submaximal contractions through shoulder flexion (proximal condition) and index finger flexion (distal condition) with the dominant arm/hand (Aune et al., 2013). Participants were first instructed to produce three unilateral MVICs with both shoulder flexion and index finger flexion. Following the three MVICs, submaximal force (25, 50 and 75%) was calculated for each participant based on the peak MVICs. Three contractions at each level of submaximal force were performed in both proximal and distal condition.

### Apparatus

**Figure 1:**
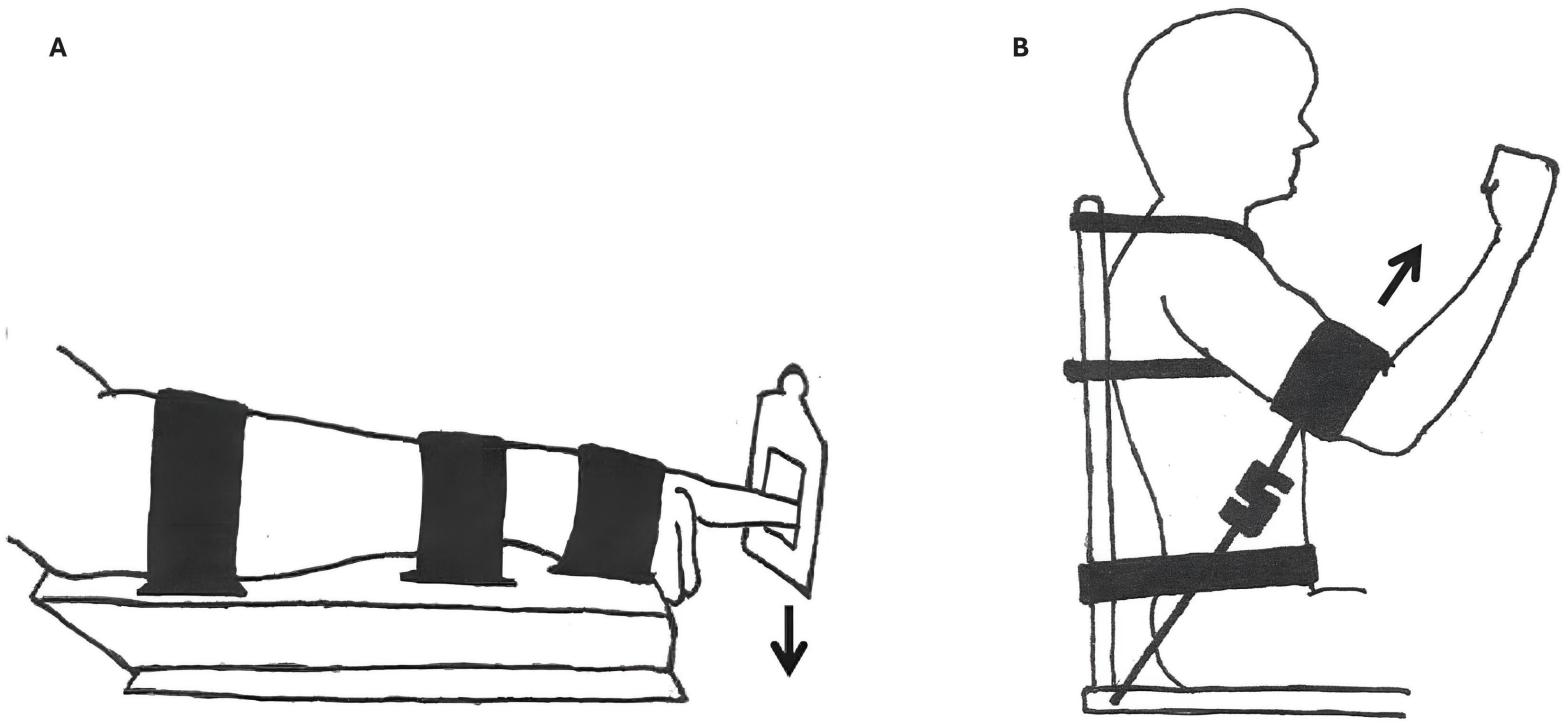
Experimental setup of the distal and proximal conditions. To constrain the participants and avoid any mechanical, postural, or synergist muscle contribution, straps and bands were used. The placement of the strap was standardized to 2 cm below the metacarpophalangeal joint on the index finger (panel **A**, distal condition) and to the lower part of the humerus 5 cm above the elbow joint (panel **B**, proximal condition). The sEMG electrodes were placed on the flexor carpi radialis (FCR; distal condition) and on the anterior section of the deltoid (proximal condition). The trunk and the arm (humerus) were set to 45° and the index finger was horizontal. The force transducers were positioned in line with the exerted force in the respective conditions (force direction is illustrated with black arrows).

A custom-made chair and apparatus were used to restrict undesired movements and reduce degrees of freedom to mechanically stabilize the participants during unilateral contractions. The intention of these constraints was to limit extraneous movement rather than to eliminate muscle contractions in non-target regions. To minimize postural instability and compensatory motion, straps and bands were applied around the waist and chest. A custom-made steel platform was used with straps and bands to constrain single degrees of freedom during index finger flexion (see Figure 1B), and for shoulder flexion, straps and bands ensured that only the targeted joint contributed to the movement (Figure 1A). Additionally, the seat height was elevated to reduce the likelihood of participants using their feet for additional support.

### Measurements

#### Surface electromyography (sEMG)

MuscleLab software (version 10.200.90.5095, Ergotest Technology A/S, Stathelle, Norway) was used to record sEMG amplitudes on the non-dominant side in the following muscles: flexor carpi radialis (FCR) for the distal condition and on the anterior deltoid for the proximal condition. For proper muscle recording, the placement of the sEMG electrodes were done according to SENIAM standardized recommendations (Konrad, 2005). Prior to placement of the self-adhesive electrodes (Dri-Stick Silver circular sEMG Electrodes AE-131, NeuroDyne Medical, Cambridge, MA, USA) with 11 mm contact diameter, 20 mm center-to-center distance on the muscles with a sampling rate of 1,000 Hz, participants’ skin was prepared to reduce skin impedance (Hermens et al., 2000; Konrad, 2005; Nazmi et al., 2016). To reduce noise, conductive gel (Signa Gel, Parker Laboratories Inc., Fairfield, NJ, USA) was applied to the electrodes. The EMG signal was recorded using the accompanying software (MuscleLab version 10.200.90.5095, Ergotest Innovation A/S, Stathelle, Norway), and the same software was used to analyze the raw EMG signals. The signals were amplified and filtered with a preamplifier near the pick-up point, bandpass-filtered (high-pass 20 Hz, low-pass 500 Hz), and then converted to root mean square (RMS) using a hardware circuit network (frequency response 450 kHz, averaging constant 12 ms; total error ± 0.5%) with a common rejection rate of 106 dB. The RMS mean was calculated for the different muscles during the isometric contractions. For normalization of the sEMG signal during the index finger flexion and shoulder flexion, the participants performed three 6-s isometric contractions (MVIC) for the muscles of interest on the non-dominant arm. The peak sEMG amplitude recorded during the MVIC, which was the same as the tasks of interest, was used to normalize the data (Besomi et al., 2020).

#### Force

Force (Newton) was measured using a transducer (S-type push-pull load cell) attached to the finger and elbow joints with static wires, sampled at 200 Hz during voluntary contractions. The transducers were aligned with the direction of the exerted force, as shown in Figure 1. Data was recorded through a data synchronization unit (DSU, MuscleLab 6,000, MuscleLab, Ergotest Innovation A/S) using the accompanying software and analyzed using a five-point differential filter in MuscleLab software (version 10.200.90.5095, Ergotest Innovation A/S).

### Procedure

The participants performed unilateral voluntary contractions with proximal (elbow flexion) and distal (finger flexion) joints on the dominant arm. The starting condition was counterbalanced across participants. Each experimental condition began with a brief task instruction, followed by three recorded MVICs and three recorded submaximal contractions at each relative force level (25, 50, and 75% of MVIC), resulting in a total of 24 voluntary contractions across both conditions. Prior to each trial, participants were given standardized instructions to keep the non-dominant arm relaxed (hanging vertically along the side of the body), avoid any compensatory movements, and produce a steady isometric contraction with the dominant arm by matching their force output to the visual target line on the screen. They were explicitly instructed to avoid co-contraction or bracing, and to maintain the required force level as consistently as possible throughout the 12-s contraction period. Each contraction was followed by a 1-min rest period to reduce fatigue.

Participants received real-time visual feedback of their force output on a screen (placed 2.5 meters in front), displayed as a continuous force trace alongside horizontal target lines corresponding to 25, 50, and 75% of their individual MVIC values. The actual relative force produced at the different submaximal force levels was for the distal condition 23.4 ± 2.2%, 47.4 ± 2.7%, and 73.2 ± 2.8% and for the proximal condition 24.3 ± 1.0%, 48.7 ± 1.5%, and 72.4 ± 2.1%.

### Data analysis

For all trials, sEMG amplitude (root mean square, RMS) was obtained from the contralateral homologous muscle on the non-dominant side (not engaged in the contraction task). Each trial lasted 12 s, and CME was quantified from the steady-state portion of the contraction. Specifically, RMS values were extracted from the 3–10 s window, as the initial and final 2 s were excluded to avoid transient influences such as task adjustments at the beginning and potential fatigue or loss of concentration toward the end (Lorås et al., 2012; Moe-Nilssen and Helbostad, 2005; Repp and Penel, 2004). The extracted RMS values were then normalized to the peak MVIC amplitude recorded from the same muscle on the non-dominant arm. These normalized RMS values served as the CME measure used in the statistical analysis.

### Statistical analysis

To determine the effect of relative force and muscles on CME, a linear mixed model with random intercept term was fitted using maximum likelihood estimation. Relative force and muscle were entered as fixed factors. Since only a random intercept was estimated, no specific assumptions about variance and correlation over time were made and the covariance structure was by default scaled identity (assumes constant variance and no correlation).

The need for random intercepts to account for heterogeneity among participants was assessed with a Wald test [see, e.g., Tang and Lin (2014)]. Although the inclusion of a random intercept term was not supported statistically [estimate = 0.072, SE = 0.044, 95% confidence interval (CI) = 0.022, 0.236, *Z* = 1.660, *p* = 0.097], the overall adjusted intraclass correlation coefficient (ICC) was 0.193, indicating that heterogeneity among participants accounted for ~19% of the total variation. Normality of residuals [see, e.g., Cheng et al. (2010)] was assessed with the Kolmogorov–Smirnov test (*D*_104_ = 0.076, *p* = 0.160) as well as visually (histogram, normal-probability plot). No values were considered outliers, determined as 3 SD outside the group mean (combination of muscle and force level).

If a significant interaction effect between relative force and muscle was present, simple effects analysis was conducted (effect of relative force per muscle, effect of muscle per level of relative force), with a Bonferroni correction for multiple comparisons. Due to the sample size, effect size was reported as Hedges’ g, derived from the differences in estimated marginal means and standard errors, and interpreted according to Cohen (2013) as trivial <0.2, small ≥0.2, moderate ≥0.5, and large ≥0.8.

Although model comparison with the intent of finding the best model was not of interest, as a formality, the fit of the unconditional model (intercept only) compared with the full model was assessed with a chi-square test using −2 Log Likelihood, with the full model showing an improvement in model fit [χ^2^ (8) = 125.8, *p* < 0.001].

All statistical analyses were performed in SPSS version 29.0.1.1 (IBM Corporation, Armonk, NY, USA). The level of statistical significance was set at *α* = 0.05.

## Results

**Figure 2:**
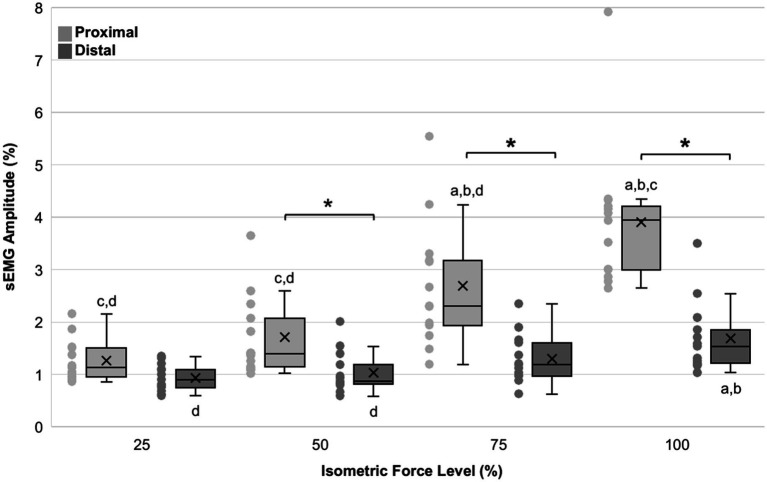
Box plots of normalized sEMG amplitude (%) in proximal (light grey) and distal (dark grey) muscles in the non-dominant arm across relative force levels (*n* = 13). The horizontal line represents the median and X represents the mean. Quartiles were calculated inclusive the median, and whiskers represent 1.5 x interquartile range outside the first and third quartile. Letters indicate significant differences (*p* < 0.05) from the respective relative force levels (*a* = 25%, *b* = 50%, *c* = 75% and *d* = 100%) within a muscle. *Indicates a significant difference between proximal and distal muscles at a given force level.

There was significant interaction between relative force and muscles in the effect on CME (F_3,91_ = 12.570, *p* < 0.001; Figure 2). For proximal muscles, simple effects analysis showed significant differences in CME with an increase in relative force (Figure 2). Pairwise comparisons indicated that CME differed between 25–75% (*p* < 0.001), 25–100% (*p* < 0.001), 50–75% (*p* < 0.001), 50–100% (*p* < 0.001), and 75–100% (*p* < 0.001), with large effect sizes (Hedges’ g 1.21–3.04).

For distal muscles, simple effects analysis also showed an increase in CME with an increase in relative force. However, the effect of force on CME for distal muscles was only significant between 25–100% (*p* = 0.004) and 50–100% (*p* = 0.019), with large effect sizes (Hedges’ g 0.81–0.93). The remaining combinations showed small effect sizes (Hedges’ g 0.12–0.48).

Between muscles, simple effects analysis showed significant differences in CME between proximal and distal muscles. Pairwise comparisons indicated that CME was significantly higher in proximal muscles compared to distal at the following relative force levels: 50% (*p* = 0.005), 75% (*p* < 0.001), and 100% (*p* < 0.001) with effect sizes ranging from moderate to large (Hedges’ g 0.76–2.50).

## Discussion

The aim of the present study was to compare and evaluate potential differences in CME for proximal (shoulder flexion) versus distal muscles (index finger flexion) in the upper extremities. The overall results showed CME for both proximal and distal muscles, with significantly higher CME observed in proximal muscles at 50, 75, and 100% of relative force levels. This difference increased with force production.

The present results confirm that unilateral motor actions are not limited to ipsilateral activation but cause CME for both proximal and distal muscles (Abreu et al., 2015; Cernacek, 1961; Cincotta and Ziemann, 2008; Heming et al., 2019; Zijdewind et al., 2006; Zijdewind et al., 1998). The observed differences between proximal and distal muscles align with known variations in bilateral neural connectivity. Cortical projections are more extensive in medial and premotor regions containing proximal representations (Brodal, 2010; Pandya and Vignolo, 1971; Gould et al., 1986), whereas distal brain areas show substantially more restricted callosal input (Jenny, 1979; Rouiller et al., 1994). Behavioral and electrophysiological studies likewise demonstrate stronger bilateral interaction and neural crosstalk for proximal than for distal effectors (Aune et al., 2020; Aune et al., 2021; Wang et al., 2022b). These converging findings indicate that proximal muscles exhibit better bilateral communication than distal muscles, which aligns with the larger CME observed for proximal muscles in our study. The current results correspond to what is observed in studies of bimanual force control, where an increase or decrease in force production by one limb can lead to a corresponding change in the force production in homologous muscles in the contralateral arm (Jin et al., 2019; Kennedy et al., 2015; Patel et al., 2019), and these results indirectly indicate a higher degree of CME with higher force produced. The present results are also in accordance with studies at a behavioral level of analysis based on the neuroanatomical and neurophysiological differences for proximal versus distal muscles, with a higher number bilateral interneurons both at the hemispheric and spinal level, showing a higher level of bilateral interference, bilateral transfer of learning, and bilateral force deficit for proximal compared to distal effectors (Aune et al., 2020; Aune et al., 2021; Aune et al., 2013; Aune et al., 2017; Škarabot et al., 2016).

Furthermore, it is interesting to observe that an increase in exerted isometric force ipsilaterally with the dominant anterior deltoid muscle (the proximal condition) causes an increase in CME in the passive (non-active), non-dominant, contralateral homologous muscle with higher relative force levels (50, 75, and 100%). In contrast, the flexor carpi radialis (FCR) muscle (the distal condition) does not demonstrate the same increase in CME with increased force and only showed a significant difference between the lower relative force levels (25 and 50%) and maximal force levels, somewhat indicating that there is a need for high neural activation to demonstrate a significant CME for distal muscles. Interestingly, when comparing CME between proximal and distal muscles across all force levels, there is a significant difference between proximal and distal muscles, where proximal muscles have a higher CME compared to distal muscles at 50, 75, and 100% of relative force levels.

The more pronounced increase in CME for proximal muscles and differences in CME between proximal and distal muscles likely reflect the greater density of commissural interneurons between the hemispheres and muscles (Brodal, 2010; Gould et al., 1986; Jenny, 1979; Pandya and Vignolo, 1971). Proximal muscles have a higher number of commissural interneurons between the hemispheres and bilateral interneurons at the spinal level compared to distal muscles. Based on the present rationale, distal muscles probably need higher ipsilateral neural activation in the working muscle to cause neural crosstalk and significant CME for homologous distal muscles. Additionally, distal muscles are also primarily activated by monosynaptic connections through the lateral corticospinal tract, which cross in the medulla oblongata, whereas proximal muscles are predominantly innervated through polysynaptic connections in the ventromedial corticospinal tract, which do not cross in the medulla oblongata (Brodal, 2010; Kuypers, 1978; Palmer and Ashby, 1992), and these differences are probably weakening the potential for neural crosstalk and CME in distal muscles compared to proximal muscles.

From a behavioral perspective, proximal muscles are more often involved in daily gross motor movements that require bilateral communication, such as maintaining postural control, performing rotational movements, or different types of locomotor behavior, such as walking and jumping (Cratty, 1962; Sorgente et al., 2021; Swift, 1903). Thus, one can assume that bilateral nerve pathways are well developed for bimanual coordination with proximal muscles, thereby promoting greater potential for CME compared to distal muscles. In addition, distal muscles are more specialized for precise and isolated movements (Baker and Wylie, 1950), reducing the need for bilateral coordination and thus resulting in a lack of increase in CME. This may be explained by the more frequent involvement of distal muscles in more isolated and precise movements that do not require the same degree of bilateral coordination. E.g., when gripping an object with the hand, there is less need for activation of muscles on the opposite side of the body.

### Limitations

Although the pronounced and progressive increase in CME observed in the proximal condition aligns with known differences in cortical and spinal connectivity supporting proximal muscle control, the present study did not measure bilateral communication and brain activity (e.g., EEG, fMRI) directly. As such, the proposed explanation – that greater cortical and spinal connectivity contributes to the stronger CME response in proximal muscles – remains inferential. Future studies combining sEMG with direct measures of brain activity are needed to verify whether the enhanced CME in proximal muscles reflects underlying differences in bilateral neural communication.

In theory, mechanical factors such as postural stability may also influence bilateral effects (Herbert and Gandevia, 1996; Zijdewind and Kernell, 2001). In the present study, although efforts were made to minimize the potential effect of stabilization demands and isolate both proximal and distal contractions through a customized experimental setup and strict instructions, the exact contribution of stabilization is ultimately unclear. However, CME was notably more pronounced in the proximal condition than the distal, suggesting that stabilization demands were not the primary cause of the observed results. Furthermore, the study did not include cross-muscle EMG recordings (e.g., anterior deltoid activity during FCR contractions and vice versa), which could have served as an additional control for unintended synergistic activation. Future studies incorporating such cross-recordings may help further verify the specificity of the observed CME patterns.

Finally, the overall sample size was relatively small, which limits the extent to which the findings can be generalized beyond young healthy adults, and the uneven gender distribution in the sample, with few female participants, prevents us from evaluating potential sex-related differences in CME. Future studies should therefore include a more balanced sex composition. Larger and more diverse samples will therefore be important for determining whether the observed CME patterns extend to broader populations.

### Practical implications and future research

The neurophysiological system plays a fundamental role in controlling and coordinating both unilateral and bilateral motor actions, and the present findings add to the understanding of how proximal and distal muscles differ in their bilateral communication. Although such knowledge about neurophysiological constraints may inform the development of training and rehabilitation strategies (Green and Gabriel, 2018; Lodha et al., 2012; Rose and Winstein, 2004; Sarabon et al., 2020; Šarabon et al., 2020; Stewart et al., 2006), the implications of our results should be interpreted cautiously. The current study was conducted in young healthy adults, and therefore the findings cannot be directly generalized to clinical populations or to rehabilitation settings. The observed pattern with higher CME in proximal muscles suggests that exercise involving proximal muscles and bilateral coordination could potentially influence CME, but this remains hypothetical. Future studies are needed to determine whether similar effects occur in other populations (e.g., older adults, individuals with neurological disorders) and whether CME can be systematically modulated through training in a way that meaningfully affects motor performance, recovery, or skill acquisition. Gaining a more detailed understanding of the neural mechanisms that produce CME would enhance the extent to which these findings can be translated into practical training and rehabilitation contexts.

## Conclusion

The aim of the present study was to compare and evaluate potential differences in contralateral muscle excitation between proximal versus distal muscles during isometric shoulder and index finger flexion. The results demonstrated CME in both muscle groups, but with significantly higher CME for proximal compared to distal muscles at the relative force levels 50, 75, and 100%. Specifically, increasing isometric force with the dominant anterior deltoid (proximal condition) was associated with a progressive rise in CME with an increase in relative force levels in the contralateral homologous muscle, whereas the flexor carpi radialis (distal condition) only showed significant CME differences between the low and maximal force levels. These findings are associated with the established neuroanatomical and neurophysiological distinctions between proximal and distal muscle representations. Proximal musculature is supported by more extensive transcallosal projections and a higher density of commissural interneurons, providing a greater capacity for bilateral neural communication. This structural and functional organization offers a plausible explanation for the stronger and more progressive CME response observed in proximal muscles. The present findings provide a foundation for future studies to advance by incorporating direct neural measures (e.g., EEG and fMRI) to further elucidate the mechanisms underlying CME.

## Data Availability

The original contributions presented in the study are included in the article/supplementary material, further inquiries can be directed to the corresponding author/s.
